# Medium Complexity Modulates Kefiran Yield and Thermal Stability in Whey-Based Fermentations: Insights from Systematic Supplementation and Comprehensive Physicochemical Characterization

**DOI:** 10.3390/polym18101168

**Published:** 2026-05-09

**Authors:** Vicente Martínez, Félix Arto-Paz, Maribel Mamani, Ricardo I. Castro, Silvana Moris, Darío M. González, Cristian Valdés

**Affiliations:** 1 Escuela de Ingeniería en Biotecnología, Centro de Biotecnología de los Recursos Naturales (CENBIO), Universidad Católica del Maule, Avda. San Miguel 3605, Talca 3466706, Chile; vicente.martinez@alu.ucm.cl; 2 Doctorado en Biotecnología Traslacional (DBT), Universidad Católica del Maule, Avda. San Miguel 3605, Talca 3466706, Chile; felix.arto@alumnos.ucm.cl; 3 Centro de Investigación de Estudios Avanzados del Maule, Vicerrectoría de Investigación y Postgrado, Universidad Católica del Maule, Avda. San Miguel 3605, Talca 3466706, Chile; maribel.viviana@gmail.com (M.M.); smoris@ucm.cl (S.M.); 4 Laboratorio Multidisciplinario de Investigación en Agroindustria, Carrera de Ingeniería en Construcción, Instituto de Ciencias Aplicadas, Universidad Autónoma de Chile, Talca 3467987, Chile; ricardo.castro@uautonoma.cl; 5 Instituto de Química, Pontificia Universidad Católica de Valparaíso (Campus Curauma), Valparaíso 2340000, Chile; dario.gonzalez@pucv.cl

**Keywords:** kefiran production, whey-based media, reducing sugars, fermentation medium, casein supplementation

## Abstract

Kefiran is a bioactive exopolysaccharide produced by kefir grains, whose synthesis is strongly influenced by culture medium composition. In this study, cheese whey was evaluated as an alternative fermentation substrate for kefiran production, and the effect of supplementation with fermentable sugars (glucose, galactose, and lactose) and casein was assessed under controlled conditions. Kefir grains were cultivated in whey- and milk-based media, and kefiran production was quantified using an anthrone-based method, while grain growth and carbohydrate consumption were monitored. Supplementation with sugars and casein reduced kefiran production by up to 34.6% and did not improve yield, whereas unsupplemented whey supported the highest kefiran concentration (86.9 ± 3.7 mg/L), comparable to that obtained in semi-skimmed milk (84.0 ± 3.0 mg/L). The recovered polysaccharide was characterized by Fourier-transform infrared spectroscopy (FTIR), proton nuclear magnetic resonance spectroscopy (^1^H NMR), X-ray diffraction (XRD), scanning electron microscopy (SEM), thermogravimetric analysis (TGA), and differential scanning calorimetry (DSC), showing structural and physicochemical properties comparable to kefiran obtained from semi-skimmed milk. These results indicate that whey constitutes a feasible and simple fermentation medium for kefiran production, and that increased medium complexity does not necessarily improve process performance.

## 1. Introduction

Kefir is a fermented beverage produced by a symbiotic consortium of lactic acid bacteria, acetic acid bacteria, and yeasts embedded in a polysaccharide matrix known as kefir grains [1]. During fermentation, this microbial community metabolizes available sugars, producing organic acids, ethanol, carbon dioxide, and exopolysaccharides (EPS) [2]. Microbial EPS are high-molecular-weight extracellular polymers widely used in the food and cosmetic industries as stabilizers and thickeners, and some also exhibit immunomodulatory and antimicrobial properties, increasing their biotechnological relevance [3,4]. Among them, kefiran is a heteropolysaccharide composed of glucose and galactose in an approximately 1:1 ratio [5], whose water solubility, gelling ability, and tunable rheological behavior enable applications in edible coatings, biodegradable films, and encapsulation systems [6]. Despite its technological potential, kefiran production traditionally relies on milk as a fermentation substrate, which increases production costs, limits achievable yields, and competes with an essential food resource, thereby constraining industrial scalability [7].

In this context, cheese whey has emerged as an attractive alternative substrate. Whey is generated in large volumes by the dairy industry, with global production estimated at approximately 160 million tons per year, as around 10 L of whey are produced per kilogram of cheese. Representing 85–95% of the processed milk volume, whey contains lactose as its main carbon source, along with soluble proteins, residual lipids, and minerals [8,9]. However, its high organic load poses environmental challenges when inadequately managed, which has driven circular economy approaches aimed at valorizing whey as a raw material for bioprocesses producing higher-value compounds [9]. Due to its abundance, low cost, and lactose content, whey has been explored as a fermentation medium for kefir cultures and EPS production. Previous studies have demonstrated that culture medium composition strongly influences kefiran synthesis, particularly the availability of carbon and nitrogen sources [10,11]. In this regard, targeted supplementation strategies have been proposed to optimize fermentation performance and enhance kefiran production [12].

From a compositional perspective, most milk proteins are caseins, which account for approximately 76–86% of total milk protein [13]. During cheese manufacture, these proteins are largely removed with the curd, and cheese whey therefore retains only a low concentration of soluble proteins, mainly β-lactoglobulin, α-lactalbumin, immunoglobulins, serum albumin, and lactoferrin [9,14]. As a result, whey-based media are relatively depleted in nitrogenous compounds. Casein supplementation has been proposed as a strategy to restore amino acid availability and enhance the viability and metabolic activity of lactic acid bacteria [15,16]. In addition, the presence of casein may influence the structure and stability of the polymeric matrix formed during fermentation [1,17]. In parallel, the nature of the carbon source plays a key role in kefiran biosynthesis. Reducing sugars such as glucose and galactose are readily assimilated by the microorganisms present in kefir grains and have been shown to promote kefiran synthesis more efficiently than lactose, which requires prior enzymatic hydrolysis [18,19]. Beyond their energetic function, these monosaccharides also serve as direct precursors for kefiran synthesis, as they are converted into activated sugar nucleotides incorporated into the glucogalactan polymer. Among them, glucose has been reported to induce higher kefiran production than other sugars, consistent with its efficient uptake and channeling into both central metabolism and EPS biosynthetic pathways [20,21]. In whey-based fermentations, total sugar concentrations around 5% (*w*/*v*) have been reported to sustain microbial growth and promote EPS accumulation without inducing detrimental osmotic effects [12].

Despite these advances, the combined effect of reducing sugar supplementation (glucose, galactose, lactose) and nitrogen restoration via casein on kefiran production in whey remains poorly understood, particularly regarding impacts on yield, purification efficiency, and functional properties beyond simple biomass metrics production [12]. While defined MRS media have achieved yields up to 1.3–2.0 g/L with pure *Lactobacillus kefiranofaciens* [22], whey-based systems, despite their industrial relevance, typically yield 50–300 mg/L due to nutritional imbalances and acid inhibition [11]. Moreover, few studies have systematically decoupled carbon source effects from nitrogen availability or linked medium composition to downstream properties like thermal decomp (T_decomp > 300 °C) and matrix organization, critical for applications in biodegradable films and bioactive delivery [23]. Therefore, this study systematically evaluates cheese whey as a fermentation substrate, assessing whether targeted co-supplementation with reducing sugars (5% *w*/*v*) and casein (3% *w*/*v*) under defined pH conditions (initial adjustment and monitoring) improves kefiran yield (>80 mg/L), structural integrity (FTIR/NMR/XRD), morphology (SEM), and thermal performance (TGA/DSC), providing actionable insights for scalable whey valorization.

## 2. Materials and Methods

### 2.1. Materials

The kefir grains used as starter culture were obtained in the Maule region (Chile). The semi-skimmed milk employed was a commercial product (Quillayes Surlat, Providencia, Chile). The whey used in this study was acid cheese whey obtained from bovine milk (Lácteos San Ignacio, Bulnes, Chile), with the following characteristics determined prior to supplementation: reducing sugar content (22.6 g/L, quantified by DNS), total protein content (8.2 ± 0.4 mg/mL, quantified by the Bradford method after appropriate dilution), initial pH (6.1), and total solids (6.2% *w*/*v*). These values are typical for acid whey derived from cheese production, where most caseins are removed with the curd, leaving a low protein content (6–10 mg/mL). Glucose (Merck, Darmstadt, Germany), galactose (Difco, Detroit, MI, USA), lactose (Merck, Darmstadt, Germany), and casein (Bacto, Detroit, MI, USA) were used as supplements. All reagents were of analytical grade.

### 2.2. Kefir Grain Growth Conditions

Prior to the fermentation experiments, reactivation was carried out by cultivating kefir grains in commercial semi-skimmed milk for seven days at room temperature, with medium renewal every 24 h. At the end of the reactivation period, the grains were drained and the wet mass was adjusted to inoculate 10 g per treatment. Grain growth was evaluated in media prepared from whey (reducing sugars: 22.6 g/L), adjusted to pH 6.1. The acidification dynamics were assessed by measuring pH every three hours for 48 h. The fermentation conditions were formulated with the following combinations and codes: whey with 5% glucose and 5% galactose (W + Gl + Ga), whey with 5% glucose and 5% lactose (W + Gl + La), whey with 5% galactose and 5% lactose (W + Ga + La), whey with 5% lactose (W + La), whey with 5% lactose and 3% casein (W + La + Ca), whey with 5% glucose, 5% lactose, and 3% casein (W + Gl + La + Ca), whey with 5% galactose, 5% lactose, and 3% casein (W + Ga + La + Ca), and whey with 5% glucose, 5% galactose, and 3% casein (W + Gl + Ga + Ca). Controls included unsupplemented whey (W), whey with 3% casein (W + Ca), and commercial semi-skimmed milk (M). The cultures were carried out in 50 mL glass flasks covered with sterile gauze to allow gas exchange, mimicking traditional kefir fermentation conditions. Each flask was inoculated with 10 g of kefir grains and incubated at 30 °C in an orbital shaker (Biobase, Jinan, Shandong, China) at 50 rpm. pH was measured every three hours for 48 h by withdrawing samples under aseptic conditions inside a laminar flow hood, with the neck of each flask flamed before and after sampling. All manipulations were performed using sterile equipment and standard microbiological techniques to prevent contamination The medium was renewed every 24 h over a period of five weeks. All whey-based culture media were sterilized by UV-C irradiation (254 nm, 15 W) for 30 min under a laminar flow hood, with constant agitation to ensure uniform exposure. Sterility was confirmed by plating on nutrient agar and incubating at 30 °C for 48 h, which showed no microbial growth prior to inoculation. All treatments were performed in triplicate.

### 2.3. Microscopy of Kefir Grain Microbiota

Gram and methylene blue staining were performed at the final cultivation time point to evaluate potential changes in the microbiota associated with kefir grains grown under the different treatments. The samples were subsequently examined using a light microscope (Leica DM500, Wetzlar, Germany) at 100× magnification. The observations were assessed qualitatively.

### 2.4. Reducing Sugars Quantification

The reducing sugars present in the samples were determined using the 3,5-dinitrosalicylic acid (DNS) colorimetric method according to Miller (1959) [24]. For each assay, 500 µL of sample was mixed with 500 µL of DNS reagent, and the mixture was incubated in a water bath at 100 °C for 5 min. The reaction was stopped by adding 10 mL of distilled water, and the absorbance was measured at 540 nm using a spectrophotometer (Shimadzu UVmini-1240, Kyoto, Japan). Concentrations were calculated by interpolation from a standard curve prepared with glucose solutions. The limit of detection (LOD) and the limit of quantification (LOQ) of the method were 0.1 mg/mL and 0.3 mg/mL, respectively.

### 2.5. Kefiran Extraction and Purification

Kefiran extraction was carried out exclusively from kefir grains collected at the end of the five-week cultivation period, as kefiran is primarily associated with the grain matrix rather than released into the culture broth [11,25]. Kefir grains, previously washed with 0.9% NaCl, were suspended in hot distilled water (80 °C) at a ratio of 1:10 (*w*/*v*) and intermittently agitated for 1 h. The mixture was centrifuged at 12,000× *g* for 20 min at 20 °C (Heraeus Megafuge 16, Thermo Fisher Scientific, Waltham, MA, USA), and the supernatant was collected. An equal volume of pre-cooled 96% ethanol was added, and the mixture was kept at −20 °C overnight. Subsequently, it was centrifuged at 12,000× *g* for 20 min at 4 °C (U-230 R, Boeco, Hamburg, Germany), and the precipitate was recovered. This precipitate was dissolved in hot distilled water (80 °C) and subjected to a second precipitation with cold denatured ethanol under the same conditions in order to reduce proteins and biomass residues. The final precipitate was dissolved in a small volume of hot distilled water and stored at −30 °C until lyophilization Lyomicron, Coolvacuum, Barcelona, Spain) for 24 h. Kefiran yield was also normalized to grain biomass and expressed as mg kefiran per g fresh kefir grains (mg/g), calculated by dividing the total kefiran mass obtained (mg) by the fresh weight of kefir grains used for extraction (g).

### 2.6. Kefiran Quantification

Kefiran quantification was performed using the colorimetric anthrone method described by Cheirsilp et al. (2001), with modifications [18]. For each assay, 0.25 mL of kefiran sample was mixed with 2.5 mL of anthrone reagent (0.8 g of anthrone dissolved in 100 mL of distilled water and 300 mL of concentrated H_2_SO_4_). The mixture was incubated at 100 °C for 10 min and then cooled to room temperature. Absorbance was measured at 620 nm using a spectrophotometer (Shimadzu Uvmini-1240, Kyoto, Japan). Kefiran concentration was calculated by interpolation from a standard curve prepared with lactose solutions. For the determinations, kefiran previously lyophilized and dissolved in distilled water was used, adjusted to a final concentration of 100 μg/mL. The limit of detection (LOD) and the limit of quantification (LOQ) were 4.7 mg/L and 15.8 mg/L, respectively.

### 2.7. Protein Quantification

Protein quantification was performed using the Bradford colorimetric method [26]. For each assay, 20 µL of sample was mixed with 980 µL of distilled water and 1 mL of Bradford reagent (Bio-Rad, Hercules, CA, USA), followed by gentle agitation. The mixtures were incubated at room temperature for 5 min, and absorbance was measured at 595 nm using a spectrophotometer (Shimadzu Uvmini-1240, Japan). Protein concentration was determined by interpolation from a standard curve prepared with bovine serum albumin (BSA) solutions. For the analyses, kefiran previously lyophilized and dissolved in distilled water was used, adjusted to a final concentration of 400 μg/mL (*w*/*v*). The limit of detection (LOD) and the limit of quantification (LOQ) were 0.119 mg/mL and 0.361 mg/mL, respectively.

### 2.8. FTIR

Lyophilized samples were analyzed by Fourier transform infrared (FTIR) spectroscopy to identify the functional groups present. Spectra were recorded in the range of 550 to 4000 cm^−1^ using a Perkin Elmer Spectrum Two spectrophotometer (PerkinElmer, Waltham, MA, USA) equipped with an attenuated total reflectance (ATR) system with a germanium crystal.

### 2.9. NMR

^1^H NMR spectra were acquired on a Bruker Avance Neo 400 MHz spectrometer (Bruker, Billerica, MA, USA). Samples were prepared in D_2_O and measured at 295 K. Chemical shifts were referenced to the residual HDO signal, adjusted to δ = 4.79 ppm.

### 2.10. SEM

The samples were analyzed by SEM with a Variable Pressure Scanning Electron Microscope (Zeiss EVO MA10, Oberkochen, Germany). The sample was coated with a ~5 nm gold layer using a Leica EM ACE200 coater (Leica Microsystems, Wetzlar, Germany). The samples were visualized at 100× magnification under high vacuum conditions.

### 2.11. XRD

Powder X-ray diffraction (PXRD) patterns were collected at room temperature using a Bruker D8 Advance diffractometer (Bruker, Billerica, MA, USA) equipped with a CuKα radiation source in a range of 5° < 2θ < 80°. Lyophilized samples were gently ground in an agate mortar prior to analysis to ensure adequate homogeneity.

### 2.12. Thermal Analysis (TGA/DSC)

The thermal stability of K samples was evaluated by thermogravimetric analysis (TGA) using a TA Instruments STD 650 analyzer (TA instruments, New Castle, DE, USA). Approximately 5.0 mg of sample was placed in a platinum crucible and heated from 50 °C to 550 °C at a constant rate of 10 °C min^−1^ under a nitrogen flow of 50 mL min^−1^. Thermal stability was assessed from the obtained TG and DTG curves. Differential Scanning Calorimetry (DSC) measurements were performed using a simultaneous DSC–TGA SDT-Q600 instrument (TA Instruments, New Castle, DE, USA). Samples were placed in an α-Al_2_O_3_ crucible and heated from room temperature (25 °C) to 550 °C. The thermal program consisted of a stabilization step at 25 °C for 1 min, followed by heating at a rate of 5 °C min^−1^. The instrument was calibrated according to the manufacturer’s specifications using sapphire as the reference material. The transition enthalpy (ΔH, J g^−1^), onset temperature (To), peak temperature (Tp), and end temperature (Tc) were determined using TA Instruments TRIOS thermal analysis software (TA Instrument-Waters LLC V 4.3).

### 2.13. Statistical Analysis

The results were expressed as the mean of triplicate values ± standard deviation. A one-way analysis of variance (ANOVA) was performed to evaluate significant differences among treatments, followed by the Tukey multiple comparison test (*p* < 0.05) to identify specific differences between groups. Statistical analyses were performed using RStudio software (version 4.5.1; RStudio, Boston, MA, USA).

## 3. Results

### 3.1. Kefir Grain Growth and Reducing Sugar Consumption

Kefir grain growth was monitored during five weeks of fermentation under eleven different culture conditions, using the percentage variation in biomass after two weeks as a growth indicator (Table 1). Medium composition had a marked effect on grain development. The Semi-skimmed milk control (M) exhibited the highest biomass increase (≈118%), which was significantly higher than all whey-based treatments (*p* < 0.05). In contrast, all whey-based media resulted in significantly lower biomass accumulation, and several formulations led to partial biomass loss (W + Gl + Ga, W + Ga + La, and W + Gl + La). Among whey-based treatments, unsupplemented whey (W) and whey supplemented with lactose (W + La) supported moderate grain growth, with no significant differences between them, whereas the addition of casein in combination with lactose (W + La + Ca) yielded the highest biomass increase within this group (36.7%). Notably, supplementation with casein alone (W + Ca) resulted in significantly reduced biomass growth compared with whey without supplementation, whereas in most sugar-supplemented media, the presence of casein mitigated biomass loss and improved growth performance.

Reducing sugar consumption measurements after 24 h of fermentation showed active substrate utilization in all treatments, with depletion values generally ranging from 60% to 80%, except for W + La, which exhibited significantly lower consumption (≈43%) compared with all other treatments (Table 2). In several formulations, including W + Gl + La, W + Ga + La, and W + Gl + Ga, high sugar depletion coincided with limited or negative biomass variation. The progressive decrease in pH observed at the end of each 24 h fermentation cycle is consistent with the accumulation of organic acids typically associated with kefir fermentation. Qualitative microscopic observations revealed Gram-positive bacilli and cocci compatible with lactic acid bacteria in all treatments, with no major differences among culture conditions, suggesting similar microbial morphology across treatments.

### 3.2. Quantification and Purity of Kefiran

Following ethanol precipitation, total protein content decreased to values below the detection limit in all treatments, and residual soluble sugars were not detected in the purified fractions, indicating effective removal of non-polysaccharidic components, with protein content below the detection limit. The reported kefiran concentrations (mg/L) correspond to the polysaccharide concentration in the final redissolved extract after lyophilization, representing a single endpoint measurement after the five-week cultivation period. These values reflect the extractable kefiran fraction recovered under the described extraction conditions (hot water, 80 °C, 1 h) and do not represent total kefiran content associated with the kefir grain matrix, as a significant portion remains structurally integrated within the grains [11,25]. The highest kefiran concentrations were obtained in unsupplemented whey (W, 86.90 mg/L) and milk (M, 83.79 mg/L), with no significant difference between these two media (Table 3). Intermediate kefiran concentrations were observed in treatments supplemented with different combinations of monosaccharides and minerals, including W + Gl + Ga, W + Ga + La + Ca, W + Ca, W + Ga + La, and W + La. In contrast, the lowest kefiran levels were detected in W + Gl + La + Ca (56.79 mg/L), followed by W + Gl + Ga + Ca, W + La + Ca, and W + Gl + La, indicating that increasing medium complexity through multiple supplements resulted in reduced kefiran production rather than enhancement. The anthrone assay provides an estimation of total carbohydrate content; therefore, the reported values represent kefiran-rich polysaccharide content rather than absolute quantification of pure kefiran. Notably, the yield of lyophilized extract (mg dry extract per g fresh grains) did not mirror the trend observed for anthrone-based kefiran quantification, suggesting that total recovered mass does not directly reflect kefiran content, while protein analysis by the Bradford method showed values below the detection limit, indicating minimal protein presence in the purified extracts.

### 3.3. FTIR Analysis

**Figure 1:**
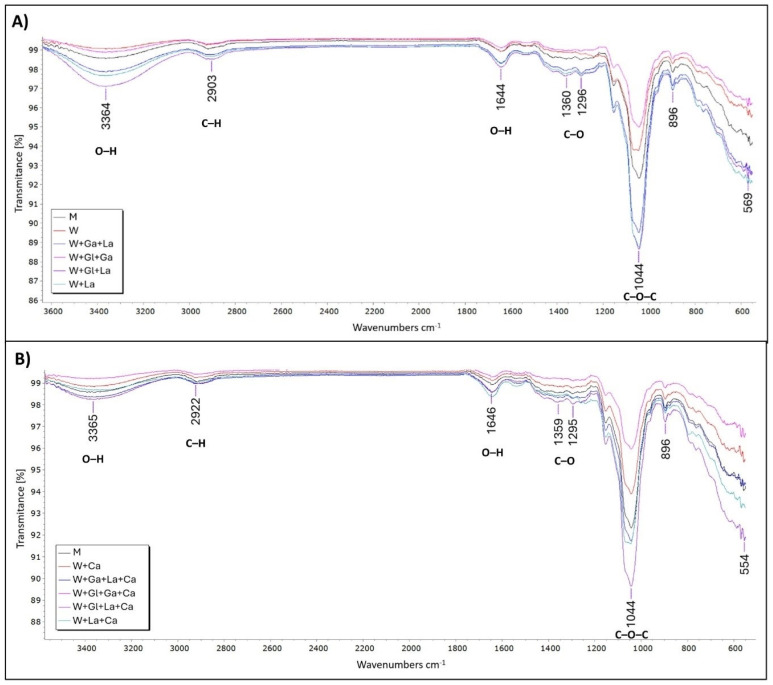
Fourier Transform Infrared (FTIR) Spectrum of the Extracted Lyophilized Kefiran. (**A**) Without casein. (**B**) With casein.

Figure 1 shows the FTIR spectra of kefiran samples obtained from all treatments, produced in media with and without casein. All samples exhibited the characteristic absorption bands of polysaccharide structures, indicating that kefiran maintained a comparable FTIR profile across all evaluated conditions. In both sets of samples, bands corresponding to O–H stretching (≈3364–3365 cm^−1^) and C–H stretching (≈2903–2922 cm^−1^) were observed, together with a signal at 1644–1646 cm^−1^ associated with O–H bending of water molecules bound to the polysaccharide matrix. Additional bands detected at approximately 1359–1360 cm^−1^ and 1295–1296 cm^−1^ correspond to C–H angular vibrations typical of polysaccharides with similar composition. The fingerprint region showed a highly consistent pattern across all treatments. In particular, the intense band at 1044 cm^−1^ corresponds to C–O–C and C–OH vibrations associated with pyranose ring structures, while the signal near 896 cm^−1^ reflects the α and β configurations of glucose and galactose monomers that define the kefiran backbone. Comparison between samples produced with and without casein revealed only minor differences in relative band intensity and in the position of low-energy peaks, such as signals around 569 cm^−1^ in samples without casein and 554 cm^−1^ in those with casein.

### 3.4. NMR Analysis

**Figure 2:**
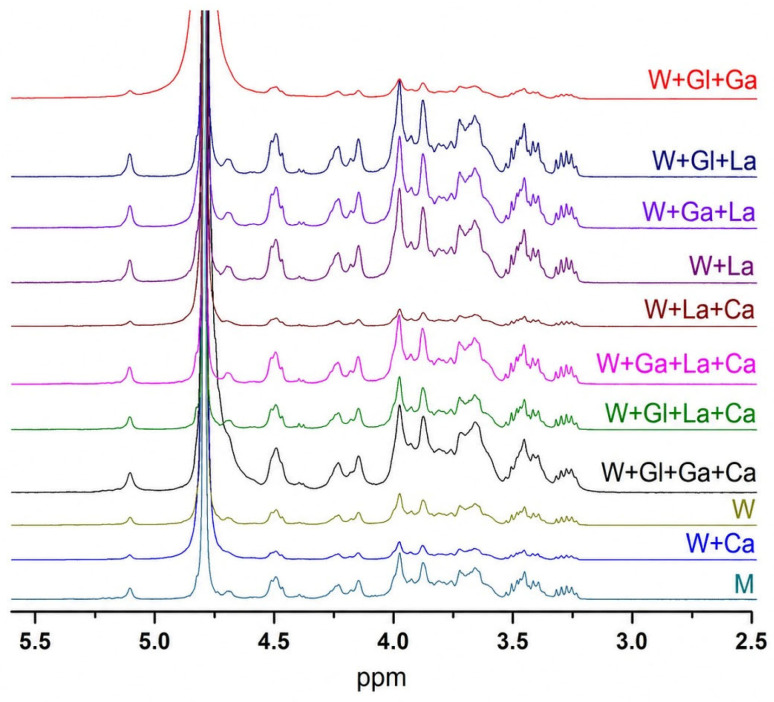
Nuclear Magnetic Resonance (NMR) spectra of the extracted lyophilized kefiran.

The ^1^H NMR spectra obtained for purified kefiran samples produced in all culture media showed an almost complete overlap, indicating a high degree of structural consistency across treatments (Figure 2). All spectra displayed resonances in the anomeric region (δ 5.3–4.5 ppm) together with a complex but reproducible pattern of signals in the sugar proton region (δ 4.3–3.0 ppm), which is characteristic of a glucogalactan polysaccharide backbone. The preservation of these spectral regions across all samples indicates that the primary structure of kefiran was maintained regardless of medium composition. No additional resonances or significant chemical-shift variations were detected between treatments that would indicate the presence of organic impurities or structural modifications of the polysaccharide. Minor differences in overall signal intensity were observed among some samples, which were attributed to variations in solubility in D_2_O rather than to changes in chemical structure, as these variations did not affect the position or relative distribution of the resonances.

### 3.5. SEM Analysis

**Figure 3:**
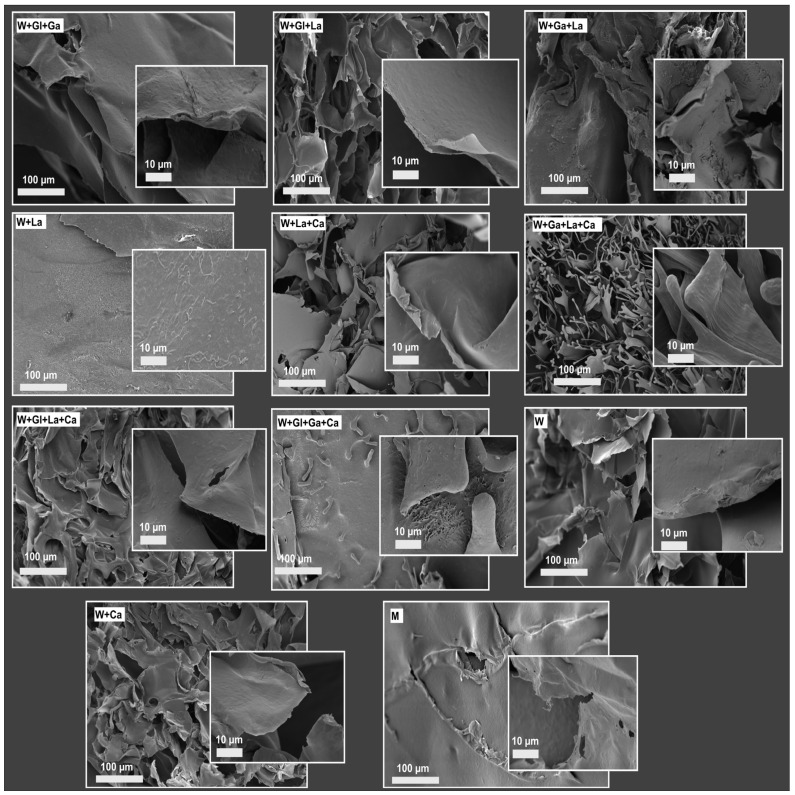
SEM images of lyophilized kefiran samples obtained under different fermentation conditions. Low-magnification images (100 µm scale) show overall morphology, while insets (10 µm scale) provide higher-magnification views. Representative images from independent samples are shown.

Scanning electron microscopy revealed that kefiran samples obtained from all eleven treatments shared a generally similar laminar morphology, which is characteristic of this exopolysaccharide, although slight variations in surface texture and compaction were observed among conditions (Figure 3). The preservation of this laminar architecture across all samples indicates that the fundamental structural organization of kefiran was maintained irrespective of culture medium composition. At low magnification (100 µm scale), all samples exhibited laminar structures with varying degrees of fragmentation and organization, whereas at higher magnification (10 µm scale), subtle differences in surface texture and microstructural features became more apparent, including variations in roughness, folding, and local porosity. In treatments without casein, kefiran exhibited clearly defined laminar structures, with relatively more uniform surfaces in glucose- and galactose-containing media and slightly increased fragmentation in lactose-based systems. Casein supplementation was associated with localized changes in matrix organization, including mild increases in compaction, folding, or microporosity, although no consistent morphological trend was observed across formulations. The control samples further illustrate the influence of medium composition on surface morphology, with unsupplemented whey (W) forming an open network of irregular laminae, whey supplemented with casein (W + Ca) showing a more continuous and densified surface, and the milk control (M) displaying the most compact and homogeneous morphology, characterized by a film-like structure with fewer visible pores.

### 3.6. XRD Analysis

**Figure 4:**
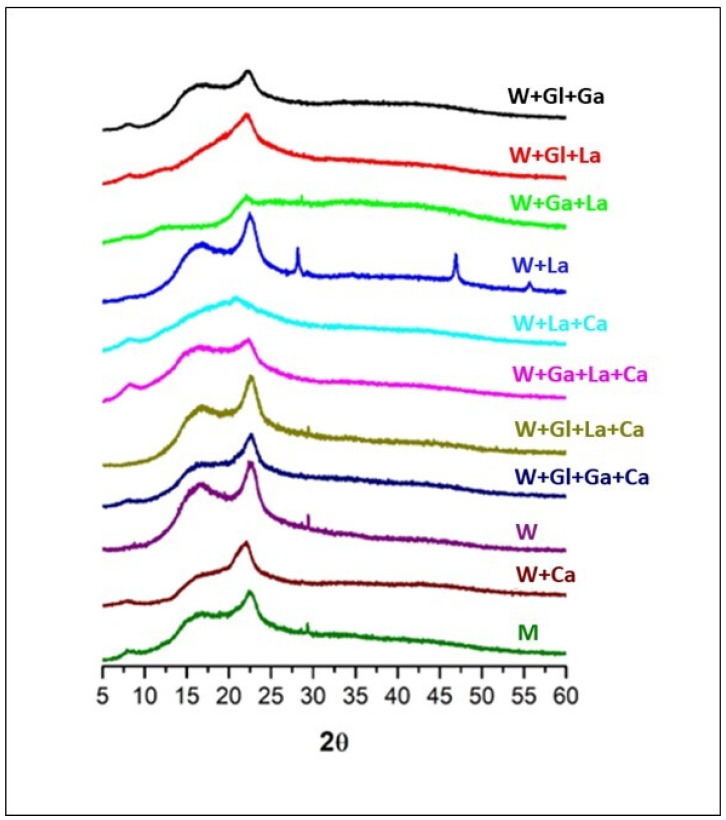
X-ray diffraction of the extracted lyophilized kefiran samples.

The X-ray diffraction patterns of kefiran powders obtained from all treatments showed a broad diffraction halo centered at approximately 2θ = 18–25°, which is characteristic of predominantly amorphous polysaccharide materials (Figure 4). The high similarity among diffraction profiles indicates that kefiran chain packing and solid-state organization were largely preserved across all culture conditions. Although no well-defined crystalline phase was detected, some samples exhibited subtle variations in the amorphous halo, such as slight narrowing or the appearance of a shoulder, suggesting minor differences in short-range molecular ordering. Isolated sharp reflections observed in certain samples were attributed to residual crystalline inorganic components rather than to the polysaccharide matrix itself.

### 3.7. TGA and DSC Analysis

The thermal behavior of kefiran samples varied depending on the culture medium, as evidenced by differences in degradation temperatures and decomposition enthalpies (Table 4). Onset degradation temperatures ranged from 286.2 °C in the milk control (M) to 306.2 °C in W + Gl + Ga, while peak decomposition temperatures extended from 298.7 °C (M) to 321.8 °C (W + Gl + Ga). Notably, the treatments with the highest sugar consumption, including W + Gl + La + Ca and M, did not yield the most thermally stable polymers, as reflected by their moderate peak decomposition temperatures. In contrast, W + Gl + Ga produced kefiran with the highest thermal stability and the highest decomposition enthalpy (ΔH = 97.1 J/g), despite exhibiting only intermediate sugar consumption. DSC analysis further showed that decomposition enthalpy provided a more sensitive descriptor of kefiran quality than temperature metrics alone. Low ΔH values, as observed for W + Ga + La, W + La + Ca, and W, suggest a matrix with higher structural heterogeneity, greater amorphous content, or the presence of non-polymeric components. In contrast, the exceptionally high ΔH measured for W + Gl + Ga supports the formation of a more ordered and cohesive polymer network under this specific fermentation condition.

## 4. Discussion

The results of this study indicate that culture medium composition influences kefir grain growth, sugar consumption, and kefiran production, while having a limited impact on the structural integrity of the exopolysaccharide. The highest grain biomass increase observed in semi-skimmed milk (M, ≈118%) is consistent with the idea that milk provides a balanced nutritional profile that optimally supports the kefir consortium, consistent with previous reports [12]. In contrast, the significantly lower biomass accumulation in whey-based media, and particularly the biomass loss in several sugar-supplemented formulations (W + Gl + Ga, W + Ga + La, W + Gl + La), suggests that carbohydrate supplementation alone may not fully compensate for the nitrogen and micronutrient deficiencies inherent to whey [7].

The finding that unsupplemented whey (W) and whey supplemented with lactose (W + La) supported moderate grain growth, with no significant differences between them, suggests that the native lactose content of whey can sustain baseline metabolic activity of the kefir grains. An important consideration for the scalability and reproducibility of whey-based kefiran production is the inherent variability of whey composition, which depends on cheese type, animal breed, diet, and processing conditions [9,14]. In this context, detailed compositional characterization of whey (including protein, fat, mineral content, and total solids) is important to improve reproducibility and enable meaningful comparison across studies. The whey used in this study is representative of acid whey, with low protein content (≈0.8% *w*/*v*) due to casein removal during cheese making. To manage feedstock variability in future studies and industrial applications, several strategies can be considered: (i) blending whey from multiple batches or suppliers to average out compositional fluctuations; (ii) using standardized whey powders or whey protein concentrates as an alternative to fresh whey; (iii) supplementing whey to a target baseline of carbon (e.g., adjusting lactose to a fixed concentration) and nitrogen sources; (iv) implementing routine quality control metrics (pH, reducing sugars, total solids, protein content) prior to fermentation to enable data normalization or real-time adjustments; and (v) documenting whey origin, processing conditions, and batch composition in all reports. These approaches would enhance process robustness and comparability across studies. However, the highest biomass increase within whey-based treatments was observed with the combination of lactose and casein (W + La + Ca, 36.7%), which may indicate that the simultaneous presence of a fermentable carbohydrate and a nitrogen source partially recreates the milk-like environment favorable for grain proliferation [12,27]. This synergistic effect is consistent with the observation that casein alone (W + Ca) resulted in reduced biomass growth compared to unsupplemented whey, underscoring that the benefit of casein may depend on the availability of an adequate carbon source. The mitigation of biomass loss by casein in sugar-supplemented media further supports the concept that kefir grain development relies on a balanced carbon-to-nitrogen ratio and could involve physical incorporation of casein micelles into the exopolysaccharide matrix [28].

The sugar consumption patterns, based on total reducing sugar depletion measured by the DNS assay, provide additional insights into metabolic partitioning. The significantly lower sugar consumption observed in W + La (≈43%) is consistent with a lower utilization of lactose as the sole carbon source in whey, compared to conditions supplemented with additional monosaccharides, possibly related to the requirement for enzymatic hydrolysis by β-galactosidase before assimilation [27]. The high sugar depletion (75–80%) observed in most supplemented treatments, coupled with limited or negative biomass variation in some cases (W + Gl + La, W + Ga + La, W + Gl + Ga), suggests that a substantial fraction of the consumed carbon was diverted toward maintenance metabolism, organic acid production, or exopolysaccharide synthesis rather than net biomass accumulation [27,28]. The consistent pH decrease across treatments is consistent with organic acid production, with lactic acid being a major contributor, as previously documented in kefir fermentation systems [4,18,29]. Microscopic observations suggested no major visible differences in microbial morphology among treatments. Gram staining enables general classification of microorganisms and has been used to identify shifts under different culture conditions; in the present study, the observed patterns were comparable across treatments, providing a general overview of the microbial structure, while more detailed characterization would require complementary analytical approaches [28].

The kefiran quantification results suggest a departure from the conventional assumption that increasing medium complexity enhances exopolysaccharide production. The highest kefiran concentrations were achieved in the simplest media: unsupplemented whey (W, 86.9 mg/L) and milk (M, 83.8 mg/L), with no statistically significant difference between them. This equivalence indicates that whey alone can act as a viable, low-cost substrate for kefiran production, capable of achieving yields comparable to the traditional milk-based system. The progressive reduction in kefiran yield with increasing supplementation, culminating in the lowest value for W + Gl + La + Ca (56.8 mg/L, a 34.6% reduction relative to W), suggests an inverse relationship between medium complexity and polysaccharide production. This phenomenon may be associated with several factors, including substrate competition among the diverse microbial consortium, osmotic stress from elevated total sugar concentrations (10–15% in supplemented media), or metabolic diversion toward lactic acid production at the expense of UDP-glucose and UDP-galactose activation for kefiran synthesis [20]. The particularly low yields in casein-supplemented sugar-poor media (W + Ca, 69.8 mg/L) may indicate that nitrogen excess in the absence of balanced carbon flux could influence EPS export or redirect nitrogen utilization toward biomass synthesis [20]. In addition, the discrepancy observed between lyophilized extract yield and anthrone-based quantification may be associated with the presence of co-recovered non-polysaccharidic components or fractions not fully detected by the colorimetric assay, which provides an estimation of total carbohydrate content, as well as the non-specific nature of the extraction process, as previously reported for kefiran, where yield does not necessarily reflect polysaccharide purity [30].

The observed yields (56.8–86.9 mg/L) are consistent with the range reported for whey-based fermentations (20–640 mg/L) [12,31] but considerably lower than those achieved in optimized defined media with pure cultures of *L. kefiranofaciens* (1.3–1.96 g/L) [22]. It should be noted that the kefiran yields reported herein (56.8–86.9 mg/L of extract) correspond to the extractable polysaccharide fraction recovered under the defined extraction conditions (hot water, 80 °C, 1 h) and do not represent the total kefiran content of the kefir grains. Complete extraction of kefiran from the grain matrix typically requires more rigorous conditions, such as prolonged heating or ultrasound-assisted extraction. Therefore, comparisons with studies reporting kefiran content as a percentage of grain dry weight should be made with caution [32]. This disparity likely reflects fundamental differences between complex kefir grain consortia and axenic cultures: in grains, a significant portion of synthesized EPS may remain associated with the biomass matrix rather than being released into the medium in a soluble, extractable form [11]. The decoupling between grain biomass increases and kefiran yield across treatments underscores the metabolic flexibility of the kefir consortium and the potential for substrate composition to redirect carbon flux without necessarily altering community structure.

The comprehensive structural characterization indicates that, despite quantitative differences in yield, the fundamental identity of kefiran was largely preserved across all culture conditions. FTIR analysis revealed the characteristic polysaccharide absorption bands—O–H stretching (≈3364 cm^−1^), C–H stretching (≈2903 cm^−1^), and the fingerprint region with the diagnostic pyranose ring vibration at 1044 cm^−1^ and the anomeric signal at 896 cm^−1^—consistent with previously reported kefiran spectra [33,34,35,36]. The minor intensity variations and slight shifts in low-energy peaks (569 cm^−1^ vs. 554 cm^−1^) between casein-supplemented and non-supplemented samples occurred outside the main structural regions and are not indicative of significant chemical modification of the polysaccharide backbone [37]. Similarly, the ^1^H NMR spectra showed near-complete overlap across all treatments, with characteristic anomeric signals (δ 4.85–4.47 ppm) and sugar proton region patterns (δ 4.3–3.0 ppm) that are hallmarks of the glucogalactan structure [12,22,30]. The absence of additional resonances confirms the purity of the extracted material and the structural stability of kefiran regardless of fermentation conditions.

SEM analysis revealed that while the fundamental laminar morphology characteristic of kefiran matrices was preserved across all samples, surface texture and organization varied depending on medium composition [23]. The more uniform surfaces in glucose- and galactose-containing media and the increased fragmentation in lactose-based systems suggest that monosaccharide composition influences the physical assembly of the polymer during drying. Casein supplementation produced localized textural changes—compaction, folding, or microporosity—without a consistent trend, indicating that protein effects are primarily physical (hydration, water retention, drying dynamics) rather than chemical [38,39]. The distinct morphologies of the control samples—open network in W, densified surface in W + Ca, and complex porous network in M—further illustrate how substrate composition can shape the physical architecture of the final polysaccharide material without altering its chemical identity [40].

XRD analysis indicated the predominantly amorphous nature of kefiran across all treatments, with the characteristic broad diffraction halo centered at 2θ = 18–25° [33]. Accordingly, the analysis was conducted for qualitative structural assessment rather than quantitative crystallinity evaluation. The subtle variations in the amorphous halo observed in some samples suggest minor differences in short-range molecular ordering, which may arise from local chain rearrangements or intermolecular interactions rather than true crystallization [41]. The isolated sharp reflections in certain patterns are likely attributable to residual inorganic contaminants rather than the polysaccharide itself, and minor differences may reflect processing history (precipitation, drying, moisture content) rather than fundamental structural alterations [42].

The thermal analysis revealed that functional properties can be modulated independently of overall yield. The highest thermal stability was achieved not in the highest-yielding medium (W) but in the glucose-galactose supplemented formulation (W + Gl + Ga), with a peak decomposition temperature of 321.8 °C and a decomposition enthalpy of 97.05 J/g. This represents an apparent enhancement compared to the milk control (298.7 °C, 74.9 J/g) and unsupplemented whey (317.8 °C, 51.4 J/g). Notably, the treatments with the highest sugar consumption (W + Gl + La + Ca, M) did not yield the most thermally stable polymers, suggesting that thermal properties depend more on the specific combination of carbon sources than on the absolute amount consumed [43]. This behavior may be associated with the influence of carbon source composition on kefiran biosynthesis, where readily metabolizable sugars such as glucose and galactose can alter metabolic fluxes and affect polymer chain organization and intermolecular interactions, thereby contributing to enhanced thermal stability [25]. The clustering of glucose- and galactose-containing formulations at higher peak decomposition temperatures suggests that the simultaneous availability of these monosaccharides promotes the formation of a more ordered polymer network with enhanced intermolecular interactions [41,44]. The exceptionally low decomposition enthalpy for W + Ga + La (17.7 J/g) indicates that not all sugar combinations are beneficial; the galactose-lactose combination appears to produce a matrix with high structural heterogeneity or amorphous content.

The dissociation between yield and thermal functionality suggests potential implications for bioprocess design. While unsupplemented whey maximizes polysaccharide quantity (86.9 mg/L), targeted supplementation with glucose and galactose (W + Gl + Ga) produces kefiran with superior thermal properties despite a slightly lower yield (77.3 mg/L). This trade-off between quantity and quality suggests that medium formulation can be strategically tailored to produce kefiran with specific functional attributes for targeted applications. For applications requiring high thermal stability—such as biodegradable films for hot-fill packaging or encapsulation of heat-labile compounds—the W + Gl + Ga formulation may be preferable despite the modest yield reduction. Conversely, for applications where quantity is the primary concern, unsupplemented whey provides the most cost-effective approach.

These findings collectively indicate that whey can serve as a versatile platform that can be rationally manipulated to produce kefiran with tunable properties. The inverse relationship between medium complexity and yield challenges the prevailing paradigm in EPS bioprocess optimization and underscores the importance of considering the complex regulatory networks within microbial consortia rather than assuming that “more is better” in medium design. The preservation of structural integrity across all conditions confirms that whey-based fermentations produce authentic kefiran, while the modulation of thermal properties through targeted supplementation opens possibilities for application-specific biopolymer production within a circular economy framework.

## 5. Conclusions

This study indicates that culture medium composition strongly influences kefir grain growth, carbohydrate consumption, and kefiran production, while having a limited effect on the structural identity of the exopolysaccharide. Semi-skimmed milk supported the highest grain biomass increase (≈118%), whereas whey-based media showed lower and more variable growth responses depending on supplementation. Notably, unsupplemented whey yielded the highest kefiran concentration (≈87 mg/L), comparable to that obtained in milk (≈84 mg/L), while additional carbohydrate and protein supplementation did not enhance kefiran production under the evaluated conditions. The lack of a direct correlation between grain biomass and kefiran yield suggests treatment-dependent metabolic responses within the kefir consortium. Thermal analyses further indicated that the functional properties of kefiran can be modulated independently of overall polysaccharide yield. In particular, supplementation with glucose and galactose (W + Gl + Ga) produced kefiran with the highest thermal stability (peak decomposition temperature ≈ 322 °C) and decomposition enthalpy (ΔH ≈ 97 J/g), despite only moderate sugar consumption. This finding indicates that medium formulation can be used to direct kefiran biosynthesis toward polymers with enhanced thermal resistance.

Structural analyses (FTIR, ^1^H NMR, XRD, and SEM) confirmed that kefiran preserved its characteristic glucogalactan structure across all culture conditions, with medium composition primarily affecting physical morphology and thermal behavior rather than the primary chemical architecture of the polymer. In this context, whey represents a viable and sustainable substrate for kefiran production. While unsupplemented whey maximized polysaccharide yield, targeted supplementation strategies may allow tuning of functional and thermal properties without compromising structural integrity, supporting the potential of whey-based processes for tailored kefiran production.

These results suggest a departure from the paradigm of “more complex = higher yield” in EPS bioprocesses, demonstrating instead that nutritional minimalism in whey unlocks maximal kefiran titers while enabling property tuning via strategic monosaccharide blends—critical for circular economy valorization of ~160 M tons/year dairy waste [9].

## Figures and Tables

**Table 1:** Percentage variation in the weight of kefir grains after 2 weeks of cultivation.

Code	Weight Variation (%)
**W + Gl + La + Ca**	20.7 ± 2.6
**M**	118.0 ± 2.5
**W + Ga + La + Ca**	16.3 ± 2.3
**W + Gl + Ga + Ca**	11.7 ± 4.7
**W**	34.0 ± 1.0
**W + Ga + La**	−5.7 ± 2.0
**W + Gl + La**	−4.3 ± 1.2
**W + Gl + Ga**	−14.0 ± 2.0
**W + Ca**	10.3 ± 1.5
**W + La + Ca**	36.7 ± 2.1
**W + La**	21.0 ± 1.5

Note: Values are expressed as mean ± SD (*n* = 3).

**Table 2:** Percentage consumption of reducing sugars after 24 h of cultivation.

Code	Sugar Consumption (%)
**W + Gl + La + Ca**	79.6 ± 2.4 ^a^
**M**	79.1 ± 2.8 ^ab^
**W + Ga + La + Ca**	78.9 ± 6.4 ^ab^
**W + Gl + Ga + Ca**	78.8 ± 6.7 ^abc^
**W**	78.5 ± 2.9 ^abc^
**W + Ga + La**	75.3 ± 3.6 ^abc^
**W + Gl + La**	73.2 ± 7.6 ^abcd^
**W + Gl + Ga**	65.9 ± 2.9 ^bcd^
**W + Ca**	65.7 ± 2.4 ^cd^
**W + La + Ca**	61.9 ± 3.6 ^d^
**W + La**	42.8 ± 4.4 ^e^

Note: Values are expressed as mean ± SD (*n* = 3). Different letters indicate significant differences (*p* < 0.05).

**Table 3:** Kefiran quantification by the anthrone method.

Code	Kefiran (mg/L)
**W + Gl + La + Ca**	56.8 ± 8.1 ^c^
**M**	83.8 ± 3.2 ^a^
**W + Ga + La + Ca**	74.7 ± 8.2 ^abc^
**W + Gl + Ga + Ca**	61.6 ± 8.8 ^bc^
**W**	86.9 ± 3.7 ^a^
**W + Ga + La**	69.0 ± 1.7 ^abc^
**W + Gl + La**	63.3 ± 8.5 ^bc^
**W + Gl + Ga**	77.3 ± 6.3 ^ab^
**W + Ca**	69.8 ± 8.4 ^abc^
**W + La + Ca**	61.8 ± 6.9 ^bc^
**W + La**	68.1 ± 7.5 ^abc^

Note: Values are expressed as mean ± SD (*n* = 3). Different letters indicate significant differences (*p* < 0.05).

**Table 4:** Values Obtained from Thermograms of the Kefiran Samples.

			TGA		DSC	
N°	Sample Code	Onset/°C	Endset/°C	Peak Max/°C	Enthalpy (ΔH) J/g	Max Temp./°C
1	W + Gl + La + Ca	296.1	327.8	306.5	81.0	311.6
2	M	286.2	322.6	298.7	75.0	297.7
3	W + Ga + La + Ca	299.5	333.2	319.9	66.6	317.1
4	W + Gl + Ga + Ca	305.1	342.2	322.4	64.6	330.2
5	w	305.5	339	317.8	51.4	328.1
6	W + Ga + La	303.6	339.2	317.5	17.7	297.1
7	W + Gl + La	295.2	329	308.9	52.6	306.2
8	W + Gl + Ga	306.2	342.4	321.8	97.1	332.6
9	W + Ca	294.7	328.8	308.1	71.1	307.7
10	W + La + Ca	302.1	333.8	314.8	20.0	316.8
11	W + La	302.8	339.3	317.5	58.0	320.7

## Data Availability

Data supporting the findings of this study are available from the corresponding author upon reasonable request.

## Abbreviations

EPS	Exopolysaccharides
FTIR	Fourier-transform infrared spectroscopy
TGA	Thermogravimetric analysis
DSC	Differential scanning calorimetry
XRD	X-ray diffraction
